# Vitabiotic: An alternative approach to diabetic foot

**DOI:** 10.1111/wrr.13222

**Published:** 2024-09-25

**Authors:** Hüseyin A. Erdem, Nazlıhan Yalçın, Arda Kaya, Meltem Taşbakan

**Affiliations:** ^1^ Department of Infectious Diseases and Clinical Microbiology Ege University Faculty of Medicine Izmir Turkey

**Keywords:** diabetic foot, micronutrients, ulcer

## Abstract

Diabetic foot ulcers and infections are complications that can result in significant morbidity such as the need for amputations, especially in uncontrolled diabetes, thereby profoundly impacting quality of life. While traditional treatments like wound care and antibiotics are effective, there is growing interest in the role of micronutrients (such as vitamins C, D, E, zinc and magnesium) in improving outcomes. This study aims to evaluate how these micronutrients affect diabetic foot infections and the need for amputation, offering insights to enhance overall prognosis. Patients who were hospitalised with a diagnosis of diabetic foot infection in the Infectious Diseases Department of Ege University Faculty of Medicine Hospital between 1 April 2022, and 31 January 2023 were included in the study. The patients' socio‐demographic information, characteristics of diabetic wounds, operation history, as well as their levels of micronutrients recorded on the case report form. A total of 202 patients were included in the study. The most common micronutrient deficiencies were vitamin D (69%), vitamin C (64%) and zinc (49%). The amputation rates were significantly higher in patients with deficiencies vitamin C, vitamin A and vitamin D (*p* < 0.005). Our research revealed a significant prevalence of vitamin deficiencies among the participants, and we observed a noteworthy correlation between amputation rates and these deficiencies. Although these findings show promise, it is essential to emphasise that micronutrient supplements should not replace traditional treatments but should rather be considered as a warning sign for preventing complications, particularly amputation or extremity loss.

**Abbreviations**
CRP: c‐reactive protein
HDL: high density lipoprotein
LDL: low density lipoprotein

## 1 INTRODUCTION

In the 19th century, it was discovered that micronutrients such as zinc, iodine and iron were crucial for maintaining good health. Several deficiency diseases were identified associated with the lack of micronutrients, including xerophthalmia, beriberi, rickets and scurvy. Empirical treatments for these diseases included consuming specific foods, such as vegetables, potatoes and citrus fruits for scurvy; milk, vegetables and meat for beriberi and cod‐liver oil for xerophthalmia and rickets. In the early 1940s, researchers began determining the necessary dietary requirements for various micronutrients. Studies showed that many people in industrialised countries suffered from multiple micronutrient deficiencies. To address this, fortified margarine, iodized salt, cod‐liver oil and flour fortification with micronutrients were introduced. As living standards improved, there was also an increase in the availability and indispensable consumption of processed foods.^1^ On the other hand, a dilemma, several studies have reported a positive relationship between improved living standards, increased consumption of energy‐dense foods and diabetes, hypertension hyperlipidaemia as metabolic syndrome. It was found that a diet high in saturated fat and refined carbohydrates, which are often consumed in high‐income countries, was associated with a higher risk of diabetes and other metabolic syndrome diseases.^2^, ^3^

Diabetic foot ulcers and infections are serious complications that can lead to significant morbidity, particularly in individuals with uncontrolled diabetes. These complications can often lead to amputations, which in turn can significantly impact an individual's quality of life. Thus, it is crucial to develop effective treatment strategies for diabetic foot complications. It is well‐known that certain micronutrients, such as vitamin C, D, E, zinc and magnesium, have positive effects on wound healing. While traditional treatments such as wound debridement, offloading and antibiotics are effective, there is increasing attentiveness to the potential role of micronutrients in improving outcomes for individuals with diabetes. This study aims to evaluate the impact of micronutrients on diabetic foot infections and wound healing, as well as their effect on the need for reoperation following amputation and overall prognosis.

## 2 MATERIALS AND METHODS

Patients who were hospitalised with a diagnosis of diabetic foot infection in the Infectious Diseases Department of Ege University Faculty of Medicine Hospital between April 1, 2022, and January 31, 2023, were included in the study after obtaining informed consent forms through voluntary participation. The patients' socio‐demographic information, diabetic wound size, presence of osteomyelitis, additional illnesses, hospitalisation/operation history, treatments received and their levels of vitamins C, D, E, B12, folic acid, zinc, magnesium and iron were recorded on the case report form.

## 3 ETHICS

The study was approved by the Local Institutional Review Board (approval no: 22‐11.1T/16, 11 November 2022).

## 4 STATISTICS

Statistical evaluations were carried out in the SAS (Version 9.4) program. Type one error (*α*) was accepted as 0.05. Chi‐square test was used to compare the proportional distributions of vitamin groups.

## 5 RESULTS

The study included a total of 202 patients (61 females, 30.2%; 141 males, 69.8%; with a mean age of 62.5 ± 6.03 years). When assessing the frequency of vitamin deficiencies among the patients, vitamin D (63/91; 69%), vitamin C (123/192; 64%), zinc (90/183; 49%), iron (84/186; 45%), vitamin A (59/190; 31%), folic acid (31/201; 15%), magnesium (16/200; 8%), B12 (13/201; 6.4%) and vitamin E (9/188; 4.7%) were ranked accordingly. The other clinical characteristics of the patients are summarised in Table 1.

**TABLE 1:** Clinical characteristics of patients.

	*n* (%)
Total	202
Gender	141 male (69.8%)
	61 female (30.2%)
Age	62.5 ± 6.03 years
HbA1c	
<6.5	51 (25.24%)
>6.5	151 (74.75%)
Wagner classification	
1/2	37 (18.3%)
3	48 (23.7%)
4	82 (40.5%)
5	35 (17.3%)
Operation type	
None	82 (40.59%)
Debridement	98 (48.51%)
Amputation	22 (10.89%)

Abbreviation: HbA1c, glycated hemoglobin.

**FIGURE 1:**
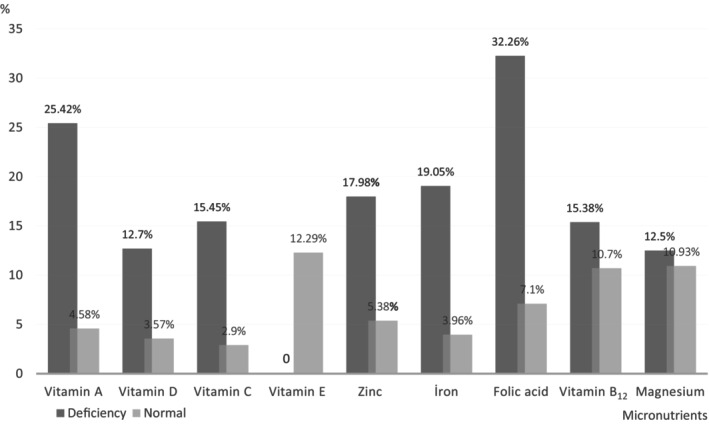
Amputation rates of the patients.

When evaluating the relationship between vitamin deficiencies and extremity amputation in patients; the amputation rate in patients with vitamin C deficiency was 15.45% (19/123), whereas in those without deficiency, it was 2.90% (2/69) (*p* = 0.0043). For those with vitamin A deficiency, the amputation rate was 25.42% (15/59), compared with 4.58% (6/131) in those without deficiency (*p* ≤ 0.0001). In patients with vitamin D deficiency, the amputation rate was 12.70% (8/63), whereas it was 3.57% (1/28) in those without deficiency (*p* = 0.0191). For patients with vitamin B12 deficiency, the amputation rate was 15.38% (2/13), compared with 10.7% (20/187) in those without deficiency (*p* = 0.8724). Patients with folate deficiency had a 32.26% amputation rate, while it was 7.10% (12/169) in those without deficiency (*p* = 0.0002). In cases of zinc deficiency, the amputation rate was 17.98% (16/89), compared with 5.38% (5/93) in those without deficiency (*p* = 0.0233). Patients with iron deficiency had a 19.05% amputation rate (16/84), whereas it was 3.96% (4/101) in those without deficiency (*p* = 0.0001). For patients with magnesium deficiency, the rates were 12.50% (2/16) and 10.93% (20/183) in those without deficiency (*p* = 0.6110). No patients with vitamin E deficiency underwent amputation, whereas the rate was 12.29% (22/179) in those without deficiency (*p* = 0.5302) (Figure 1).

**FIGURE 2:**
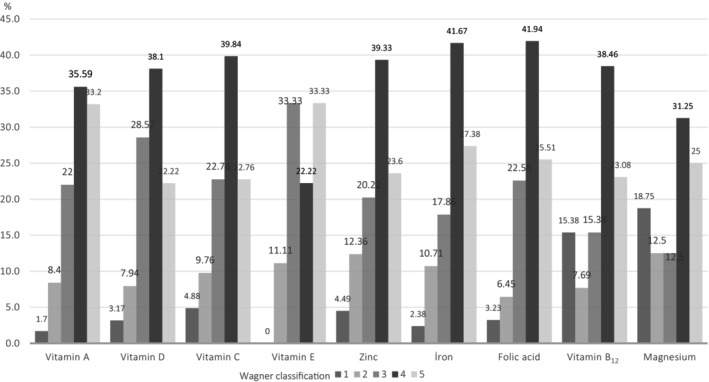
Wagner classification and the patients with vitamin deficiency.

Wagner classification and vitamin deficiencies was evaluated, among patients with Wagner grade 5 ulcers, the prevalence of vitamin A deficiency was 32.20% (19/59), compared with 10.69% (14/131) in those without deficiency (*p* = 0.0023). Similarly, among patients with vitamin C deficiency, the prevalence of Wagner grade 5 ulcers was 22.76% (28/123), whereas it was 5.8% (4/69) in those without deficiency (*p* = 0.007). Furthermore, the prevalence of Wagner grade 5 ulcers among patients with iron deficiency was found to be 27.38% (23/84), compared with 11% (11/100) in those without deficiency (*p* = 0.0027). No statistically significant differences were observed in vitamin deficiencies between HbA1c level, Wagner classifications^1^, ^2^, ^3^, ^4^ among the patients. Wagner classification and the patients with vitamin deficiency is shown in Figure 2.

## 6 DISCUSSION

Diabetic foot ulcers are a serious clinic condition that can easily develop, but they are difficult to treat, monitor and manage. Especially in high‐risk patients, vascular, neurological and metabolic disorders should be carefully investigated in terms of preventing ulcer development, wound healing and amputation. In addition to the known classical etiological reasons for the formation of diabetic foot ulcers, knowing the levels of micronutrients can be beneficial in preventing the severity of the disease and complications. Pena et al. evaluated 131 patients with diabetic foot ulcers to determine the prevalence of micronutrient deficiencies in their prospective study. The most common micronutrient deficiencies found were vitamin D (55.7%), vitamin C (50.8%), zinc (26.9%), vitamin A (10.9%) and ferritin (5.9%), respectively. The study statistically proved a significant correlation between the severity of diabetic foot ulcers and vitamin C deficiency, suggesting that healthcare professionals should assess the levels of micronutrients in patients with diabetic foot ulcers and consider supplementation if a deficiency is found.^4^ We observed a high incidence of vitamin deficiencies in the patients included in our study. Although the higher prevalence of vitamin deficiencies in a disease accompanied by intense systemic metabolic irregularities, such as diabetes, is an expected finding, it can still be a valuable outcome in monitoring such patients and anticipating potential complications.

Several studies have investigated the potential benefits of micronutrient supplements in treating diabetic foot complications. Razzaghi et al. evaluated the effect of vitamin D supplementation on wound healing in a randomised controlled study. Sixty patients with third‐degree foot ulcers according to the Wagner–Meggitt classification were included and compared with a placebo group for 12 weeks. The group receiving vitamin D supplementation achieved a significant decrease in ulcer size (−2.1 ± 1.1 vs. −1.1 ± 1.1 cm, *p* = 0.001), width (−2.0 ± 1.2 vs. −1.1 ± 1.0 cm, *p* = 0.02) and depth (−1.0 ± 0.5 vs. −0.5 ± 0.5 cm, *p* < 0.001). Additionally, it is thought that vitamin D supplementation may have a positive contribution to wound healing by achieving a reduction in low‐density lipoprotein (LDL), total/high‐density lipoprotein (HDL) cholesterol levels and providing glycaemic control in patients.^5^ Another randomised controlled study, planned with a similar method by Razzaghi et al., investigated the effect of magnesium supplementation on wound healing in diabetic foot ulcers. A total of 70 patients with grade 3 foot ulcers were included in the study and were compared the group receiving daily 250 mg magnesium oxide supplementation for 12 weeks with the placebo group. It was reported that there was a statistically significant improvement in ulcer size, blood sugar regulation, C‐reactive protein (CRP) level and plasma antioxidant capacity in the patient group receiving magnesium supplementation.^6^ Momen‐Heravi et al. conducted a randomised, placebo‐controlled, double‐blind study to investigate the effect of zinc supplementation on wound healing in diabetic foot ulcer patients with grade 3 ulcers. Sixty patients (30 participants in each group) were given 50 mg elemental zinc daily for 12 weeks. The study reported that zinc supplementation had a positive effect on ulcer size (length [−1.5 ± 0.7 vs. −0.9 ± 1.2 cm, *p* = 0.02] and width [−1.4 ± 0.8 vs. −0.8 ± 1.0 cm, *p* = 0.02]), serum insulin concentration (−8.0 ± 15.4 vs. +1.1 ± 10.3 μIU/mL, *p* = 0.009) and other similar metabolic variables in the group receiving zinc supplements.^7^ In another randomised, placebo‐controlled study by a similar working group, the effect of magnesium and vitamin E co‐supplementation on the healing of diabetic foot ulcers was evaluated. Fifty‐seven patients were included (intervention group *n* = 29, placebo *n* = 28) and were given 250 mg of magnesium oxide plus 400 IU of vitamin E daily for 12 weeks. As a result of study, compared with placebo, magnesium and vitamin E co‐supplementation had beneficial effects on ulcer size, glycaemic control, triglycerides, levels in patients with diabetic foot ulcer.^8^ In our study, we could not find significant results in the vitamin E and magnesium deficiency group among patients who experienced extremity loss due to amputation, unlike other micronutrients. However, we believe it would be incorrect to conclude that replacing these micronutrients does not help in wound healing or in preventing/reducing extremity loss based on our finding. Along with studies demonstrating the effectiveness of micronutrients, The International Working Group on the Diabetic Foot guide recommends not using pharmacological agents that supplement vitamins and trace elements to improve wound healing. This recommendation is given a strong grade but with low quality of evidence.^9^

The main limitation of our study is the descriptive design, which specifically results in the lack of evaluation of the effect of replacement therapy on wound healing and prevention of potential reoperation or amputation needs, especially in the group of patients with vitamin deficiencies who did not undergo debridement or surgical intervention. Another important limitation and the disadvantage of the retrospective design of our study, which made it impossible to access complete vitamin data for all patients. Some vitamin tests could not be performed at our hospital at that time due to infrastructure limitations, reimbursement and billing issues or because the clinician may have forgotten to request the test. This situation necessitated the evaluation of a different number of patients for certain vitamins. Another limitation of our study is that only the Wagner classification was used for the assessment of diabetic foot.

Despite these promising findings, micronutrient supplements should not be used as a replacement for traditional treatments but rather as complementary therapy. Further studies are needed to determine the safety and efficacy of combining micronutrient supplements with other treatments for diabetic foot complications. Monitoring the micronutrient levels of patients may be an important consideration in preventing complications, especially amputation/extremity loss, which often occur in conjunction with many metabolic disorders, particularly diabetic foot ulcers and infections, in diabetic patients.

## CONFLICT OF INTEREST STATEMENT

The authors declare no conflicts of interest.

## Data Availability

The data that support the findings of this study are available on request from the corresponding author. The data are not publicly available due to privacy or ethical restrictions.
